# Specific Interaction of TMEM9A with Neural Endosomal Antiporters ClC-3 and ClC-4

**DOI:** 10.3390/cells15211932

**Published:** 2026-10-24

**Authors:** Eugenia Rubino, Abraham Tettey-Matey, Alice Giusto, Francesca Sbrana, Margherita Festa, Maria Antonietta Coppola, Cristiana Picco, Paola Gavazzo, Elena Angeli, Michael Pusch, Raffaella Barbieri

**Affiliations:** 1 Istituto di Biofisica, Consiglio Nazionale delle Ricerche, 16149 Genova, Italy; eugenia.rubino@ibf.cnr.it (E.R.); abraham.matey@ibf.cnr.it (A.T.-M.); alice.giusto@ibf.cnr.it (A.G.); francesca.sbrana@ibf.cnr.it (F.S.); margherita.festa@edu.unige.it (M.F.); mariaantonietta.coppola@ibf.cnr.it (M.A.C.); cristiana.picco@ibf.cnr.it (C.P.); paola.gavazzo@ibf.cnr.it (P.G.); raffaella.barbieri@ibf.cnr.it (R.B.); 2 Current addresses: DISTAV, Dipartimento di Scienze della Terra, dell’Ambiente e della Vita, Università di Genova,16132 Genova, Italy; 3 DIFI Lab, Dipartimento di Fisica, Università di Genova, 16146 Genova, Italy; elena.angeli@unige.it

**Keywords:** neurodevelopmental disorder, accessory subunit, endosome, chloride transport, CLC proteins

## Abstract

**Highlights:**

**What are the main findings?**

• TMEM9A selectively inhibits the endosomal CLC transporters ClC-3 and ClC-4. In contrast, TMEM9A has no detectable effect on ClC-2 or ClC-7 and only a modest effect on ClC-1, showing a high functional specificity within the CLC family.
• TMEM9A directly associates with ClC-3 and ClC-4. Fluorescence lifetime imaging of Förster resonance energy transfer (FLIM-FRET) measurements confirm close molecular interaction with both transporters, consistent with the electrophysiological inhibition. Tmem9a, Tmem9b, Clcn3, and Clcn4 show overlapping expression in the brain. TMEM9A partially colocalizes with ClC-3 in cultured primary neurons of mice.

**What are the implications of the main findings?**

• TMEM9A is a specific accessory β-subunit of the neuronal endosomal CLC transporters. Together with previous studies of TMEM9B and the recent structural and genetic evidence, the findings support a model in which TMEM9A proteins selectively regulate the highly similar ClC-3/ClC-4/ClC-5 subgroup rather than CLC proteins in general.
• TMEM9A and TMEM9B may constitute a partially redundant regulatory system controlling overactive endosomal chloride/proton exchange in the nervous system. Their overlapping brain expression and similar effects on ClC-3 and ClC-4 provide a basis for understanding the severe phenotypes associated with combined loss of TMEM9A/TMEM9B inhibition and suggest that altered β-subunit regulation could contribute to disorders caused by dysregulated endosomal CLC activity.

**Abstract:**

Endosomal ClC-3 and ClC-4 Cl^−^/H^+^ antiporters are essential regulators of vesicular ion homeostasis and neuronal function, yet the mechanisms controlling their activity remain incompletely understood. TMEM9A has recently emerged as an accessory β-subunit of endosomal CLC transporters. Here, we define the functional and molecular specificity of TMEM9A across the CLC family. Electrophysiological recordings in Xenopus laevis oocytes and HEK293 cells demonstrated that TMEM9A strongly suppresses ClC-3- and ClC-4-mediated transport, while producing no detectable inhibition of ClC-2 or ClC-7 and only a modest effect on ClC-1. Consistent with these functional effects, colocalization microscopy and FLIM-FRET revealed close molecular association of TMEM9A with ClC-3 and ClC-4, with FRET efficiencies of 0.19 ± 0.02 and 0.15 ± 0.02, respectively, whereas substantially weaker interactions were detected with ClC-1 and ClC-7. In the mouse brain, Tmem9a, Tmem9b, Clcn3, and Clcn4 displayed broad and overlapping expression across cortex, hippocampus, and cerebellum. Moreover, immunofluorescence analysis showed moderate colocalization of TMEM9A with ClC-3 in primary neurons and substantial colocalization with ClC-3 and ClC-4 in heterologous cells. Together, these findings establish a preferential functional and molecular association of TMEM9A with ClC-3 and ClC-4, closely mirroring the selectivity of TMEM9B, and support the TMEM9 proteins as a regulatory β-subunit system for the neuronal endosomal CLC transporters.

## 1. Introduction

The nine members of the CLC (chloride channel—Cl^−^/H^+^ antiporter) family play fundamental roles in anion and proton transport in mammalian cells. They include plasma-membrane localized chloride channels (ClC-1, ClC-2, ClC-Ka, ClC-Kb) and intracellular endosomal/lysosomal 2 Cl^−^/1 H^+^ antiporters (ClC-3, ClC-4, ClC-5, ClC-6, ClC-7). Mutations in each of the CLC genes lead to a variety of inherited human diseases —ranging from myotonia congenita (ClC-1), leukodystrophy (ClC-2), Bartter syndrome (ClC-Ka/-Kb), Dent’s disease (ClC-5), osteopetrosis (ClC-7), neuro-developmental delay and neurodegeneration (ClC-3, ClC-4, ClC-6, ClC-7)—underscoring the physiological importance of this family [1,2,3,4].

The vesicular CLC proteins ClC-3 to -7 have emerged as pivotal regulators of organellar ion homeostasis, membrane potential, and vesicular trafficking. They play critical roles in the acidification and ionic balance of endolysosomal compartments—functions that extend beyond mere pH control to establishing Cl^−^ flux as a regulatory signal in endosome biology [1,5,6].

While the pore-forming CLC subunits are responsible for basic ion transport activity, their physiological behavior, localization, and stability are often dictated by accessory or auxiliary proteins. Accessory proteins of CLCs serve multiple critical roles, including trafficking, membrane targeting, gating modulation, and degradation control. For instance, the β-subunits barttin (BSND) and OSTM1 are essential partners of the kidney and lysosomal CLC isoforms, respectively. Barttin binds to ClC-Ka and ClC-Kb, enabling their proper insertion into the plasma membrane and regulating channel gating. Mutations in BSND cause Bartter syndrome type IV, underscoring the importance of this interaction [7,8,9]. Similarly, OSTM1 forms a stable complex with ClC-7, necessary for its lysosomal localization and protection from proteolytic degradation. Loss of OSTM1 leads to osteopetrosis and neurodegeneration due to defective lysosomal function [10,11,12,13]. The interaction of ClC-2 with the single-span transmembrane protein GlialCAM is essential in astrocytes and oligodendrocytes, but not in other cell types, where *GLIALCAM* is not expressed [14,15,16]. Mutations in *GLIALCAM* cause megalencephalic leukoencephalopathy with subcortical cysts, a leukodystrophy characterized by early-onset macrocephaly and delayed-onset neurological deterioration [16].

Until recently, no accessory protein was known for the endosomal branch comprising the highly similar proteins ClC-3, ClC-4, and ClC-5. In 2024, Festa et al. described for the first time that the single-pass transmembrane protein TMEM9B directly interacts with ClC-3 and ClC-4, and inhibits their transport activity [17]. This provided the first evidence that the sub-family of endosomal CLCs can be regulated by small β-subunit proteins. Following this, Planells-Cases et al. [18] uncovered the broader importance of β-subunits (both TMEM9A and TMEM9B) in vivo: they showed that TMEM9A and TMEM9B are obligatory β-subunits for ClC-3, ClC-4 and ClC-5, and that mice lacking both genes suffer embryonic or perinatal lethality and severe reduction in the three transporter proteins, suggesting they also offer transporter stability. Most recently, Schrecker et al. [19] provided the structural basis of this regulation: cryo-EM structures showed how TMEM9A binds to ClC-3, sealing the cytosolic entrance to the Cl^−^ pathway, and revealed that the phosphoinositide PtdIns(3,5)P_2_ stabilizes the TMEM9A–ClC-3 complex and is required for proper inhibition of the transporter.

Taken together, these studies paint a coherent emerging picture: accessory single-pass proteins (TMEM9A/9B) join the endosomal CLC antiporters as bona fide regulatory β-subunits, critical for their proper functional repression, stability, and physiological homeostasis of endosomal membranes. Yet important questions remain. First, although the specificity of TMEM9B for ClC-3/4 has been demonstrated by Festa et al. [17], and in vivo roles of both TMEM9A/9B have been shown by Planells-Cases et al. [18], the precise transport-inhibitory profile and binding specificity of TMEM9A (as opposed to TMEM9B) remain to be characterized in heterologous expression systems. Second, the work by Schrecker et al. focused on ClC-3 exclusively but not other CLC family members (ClC-1, ClC-2, ClC-7, etc.) for specificity of functional impact. Finally, co-localization of TMEM9 proteins in situ with CLC proteins has not yet been demonstrated. Thus, a systematic functional and interaction-specific study of TMEM9A across multiple CLC family members is warranted.

In this study, we address these gaps. Our results show that TMEM9A strongly suppresses ClC-3 and ClC-4 current, with only minimal or no effect on ClC-1, ClC-2, or ClC-7, and confirm, by means of FLIM-FRET, that TMEM9A physically binds to ClC-3 and ClC-4, but not to the other CLCs. Moreover, a broad and overlapping expression of Clcn3, Clcn4, Tmem9a, and Tmem9b is detected in mouse brain, and interestingly, in cortical neuronal cultures a moderate degree of co-localization with ClC-3 is observed. These findings suggest that TMEM9A exhibits a selectivity profile virtually matching that of TMEM9B, supporting the conclusion that both accessory proteins share overlapping physiological functions and are likely to form a regulatory module for the endosomal CLC antiporter sub-family.

By defining the interaction and inhibitory specificity of TMEM9A across the CLC family, our study advances understanding of endosomal ion-transport regulation and sets the stage for future investigations into how dysregulation of these β-subunits may contribute to disease phenotypes of CLC transporter mutations.

## 2. Materials and Methods

### 2.1. Expression Constructs

ClC-1, ClC-2, ClC-3, ClC-4, and ClC-7 constructs were the same as those described previously [17]. For generation of the mouse TMEM9A-mCherry construct, the Mus musculus TMEM9A open reading frame was amplified from pLV Tmem9-V5 (Addgene plasmid #175151) using the primers TMEM9A-HindIII (atgaagcttatgaagctgctgtgtttggtggc) and TMEM9A-NotI-R (tggcggccgcactgagcatcttgtgtcg). The resulting PCR fragment was digested with HindIII and NotI and ligated into the corresponding sites of the dephosphorylated pFrog-mCherry vector [20]. For expression in Xenopus oocytes, the TMEM9A coding sequence was additionally subcloned into the pTLN vector [21]. All recombinant plasmids were confirmed by Sanger sequencing.

### 2.2. The Oocyte Expression System

For in vitro synthesis of cRNA and its subsequent expression in oocytes, the pTLN plasmid was first linearized using the MluI restriction enzyme (Thermofisher, Milan, Italy). Successful linearization of the DNA templates was confirmed by agarose gel electrophoresis. Subsequently, RNA was transcribed using the mMESSAGE mMACHINE SP6 Transcription Kit (Thermofisher, Milan, Italy). Following synthesis, integrity and quality of RNA were verified on agarose gel. The final samples were then stored at −20 °C. Xenopus laevis oocytes were injected with 2.5 ng of ClC-1, 8 ng of ClC-4 and 0.8 ng of TMEM9A cRNA, then incubated at 18 °C for 2–3 days before measurements. In experiments with ClC-1, the expression of TMEM9A was verified by parallel co-injection of TMEM9A with ClC-4, which always resulted in efficient inhibition of ClC-4 currents.

### 2.3. Two-Electrode Voltage-Clamp

Two-electrode voltage-clamp recordings of ClC-1 and ClC-4, expressed with or without TMEM9A, were performed in Xenopus oocytes at room temperature (20–22 °C). The recording solution contained 100 mM NaCl, 10 mM Hepes, and 5 mM MgSO_4_ (pH 7.3). Data were acquired using the freeware acquisition program GePulse (http://users.ge.ibf.cnr.it/pusch/programs-mik.htm (accessed on 1 August 2023)).

Ionic currents mediated by ClC-4, expressed either alone or together with TMEM9A, were recorded using a voltage-clamp protocol consisting of 19 depolarizing pulses (10 ms each) applied from a holding potential of −30 mV. Test potentials ranged from +170 mV to −10 mV in 10 mV decrements. The identical protocol was applied to uninjected oocytes, and their currents were subtracted from those recorded in injected cells following baseline correction. Current amplitudes were normalized to the value measured at +170 mV for the corresponding wild-type transporter expressed without TMEM9B. Data represent the mean value of four independent injection experiments, with at least six oocytes analyzed for each construct in every injection. For improved visualization, residual capacitive transients were omitted from the displayed current traces.

ClC-1 currents were measured using a protocol comprising an initial 100 ms pulse to +60 mV, followed by thirteen 200 ms voltage steps from −160 mV to +80 mV in 20 mV increments, and ending with a 100 ms pulse to −100 mV. Between voltage-clamp sweeps, the membrane potential was maintained at −40 mV. Data are shown as the average of three independent injections, each including recordings from at least six oocytes. Expression levels were estimated from the slope conductance of the steady-state current–voltage relationship between −40 and +40 mV, the voltage range exhibiting the maximal conductance slope. This parameter provides the most reliable measure of ClC-1 expression levels [20].

All electrophysiological recordings from Xenopus oocytes were performed 48–72 h after cRNA injection.

### 2.4. Patch Clamp

Whole-cell patch-clamp recordings were performed on HEK293 cells transiently expressing CLC family members either alone or in combination with TMEM9A at a ratio of 2:1 (300ng ClC/150ng TMEM9A, fused to mCherry), as previously described [6,17]. The expression of TMEM9 was verified for each patch-clamped cell by the presence of mCherry fluorescence. Recording pipettes were pulled from borosilicate glass capillaries (Hilgenberg, Malsfeld, Germany) and had resistances of 1.8–2.5 MΩ when filled with a standard intracellular solution containing (in mM): 130 NaCl, 10 Hepes, 2 MgCl_2_, and 2 EGTA (pH 7.3). The standard extracellular solution contained (in mM): 160 NaCl, 10 Hepes, and 4 MgSO_4_ (pH 7.4). A 3 M KCl/1% agar bridge served as ground.

Electrophysiological recordings were filtered at 10 kHz with the built-in 8-pole Bessel filter of an Axopatch 200 amplifier (Molecular Devices, San Jose, CA, USA) and sampled at 100 kHz. Data were acquired via a National Instruments interface (NI PCI-6036, Austin, TX, USA) using GePulse software(accessed on 1 August 2023). Analysis was carried out using Ana. Both programs are freely available at http://users.ge.ibf.cnr.it/pusch (accessed on 1 August 2023). Additional data analysis was conducted using Sigmaplot (Jandel Scientific, San Rafael, CA, USA) where appropriate.

The specific protocols used to characterize the current–voltage relationship of each CLC member have been previously described [4,15,22] and are briefly summarized here as follows: to record ClC-3 transport currents, 5 ms voltage pulses were applied from a holding potential Vhold of 0 mV, decreasing from +200 to −40 mV in 10-mV steps. Capacitive and leak currents were removed from raw measurements via a P/N subtraction procedure utilizing a 0.2× scaled measurement protocol, followed by appropriate scaling and subtraction of the residual currents. To assess ClC-2 activity, the protocol used consisted of holding the membrane at a Vhold of 0 mV followed by a prepulse to 60 mV for 250 ms. Subsequently, a 2 s test pulse ranging from −120 mV to 60 mV was applied, followed by a 250 ms pulse at 60 mV before returning to 0 mV. With respect to ClC-7 biophysical measurements, the protocol we employed consisted of a holding potential of 0 mV, from which 500 ms activating test pulses were delivered from +140 mV to −80 mV in 20 mV decrements, followed by a constant tail pulse at −80 mV.

### 2.5. FLIM-FRET and Fluorescence Confocal Imaging

A Stellaris 8 Falcon τ-STED microscope (Leica Microsystems, Mannheim, Germany) equipped with an 80 MHz supercontinuum white-light pulsed laser (440–790 nm) and an HC PL APO CS2 100×/1.40 oil-immersion objective was employed for both confocal fluorescence imaging and FLIM-FRET experiments. Fluorescence intensity images and lifetime information were recorded simultaneously during laser scanning using a frame size of 512 × 512 pixels, a pixel dwell time of 3.16 μs, and 16-line accumulation. GFP, used as the donor fluorophore, was excited at 488 nm, and its emission was collected between 502 and 558 nm with a hybrid detector operating in photon-counting mode (Leica Microsystems, Mannheim, Germany). The same acquisition parameters were employed for conventional GFP imaging. For mCherry imaging, excitation was provided at 561 nm and fluorescence emission was detected in the 571–628 nm range.

Fluorescence lifetime analysis was carried out using the fit-free phasor approach [23]. For each pixel, the fluorescence decay profile was converted into frequency-domain information by Fourier transformation, generating two phasor coordinates, G and S, corresponding to the cosine and sine components of the fluorescence response. The distribution of these coordinates was represented as a two-dimensional density map in the G–S plane, where the signal density reflected the number of pixels sharing similar phasor values. The dominant pixel population was identified by Gaussian fitting, and the corresponding fluorescence lifetime was calculated according to:

(1) $$\tau =\frac{S}{2\pi \mathrm{fG}}$$

where *f* = 80 MHz is the laser repetition frequency.

Phasor-based FLIM-FRET analysis was performed using the custom AnaVision software (http://users.ge.ibf.cnr.it/pusch/programs-mik.htm; accessed on 15 February 2024). Regions of interest (ROIs) were selected either along the plasma membrane or within intracellular regions displaying intermediate fluorescence intensity, while excluding areas with obvious protein aggregation or saturated signal. The ROIs shown in the figures were restricted to the plasma membrane, but no appreciable differences were observed between the two cellular regions.

Förster Resonance Energy Transfer (FRET) efficiency, E, is determined by the spectral overlap between donor emission and acceptor absorption, as well as by the distance and relative orientation of the fluorophores. FRET was quantified from the reduction in donor fluorescence lifetime, comparing the lifetime measured in the absence of the acceptor (τ_D_) with that measured in its presence (τ_DA_), according to:

(2) $$E=1-\frac{\tau_{\mathrm{DA}}}{\tau_{D}}$$

For each experimental session, τ_D_ was determined using cells expressing only the donor fluorophore. Fluorescence lifetime measurements from at least three independent transfection experiments were pooled for the final analysis.

### 2.6. ClC-3 Venus Mouse Model

Since commercially available ClC-3 antibodies do not work well in immunohistochemistry, we exploited the knock-in Clcn3ven/ven mouse model, in which the Venus tag is fused to the N-terminus of the ClC-3 protein, and which is indistinguishable from WT mice [24]. These mice were kindly provided by Thomas Jentsch (FMP, Berlin, Germany).

### 2.7. Cortical Neuronal Cultures

Primary cortical neurons were prepared from embryonic day 14 (E14) C57BL/6 Venus mouse embryos. Pregnant dams were euthanized by CO_2_ inhalation at gestational day 14, and the embryos were rapidly collected and transferred into ice-cold calcium- and magnesium-free Hank’s Balanced Salt Solution (HBSS; Thermo Fisher Scientific, Waltham, MA, USA). The brains were carefully dissected by removing the skin and skull to isolate the cerebral cortices. Cortical tissue was mechanically dissociated in HBSS using a series of fire-polished Pasteur pipettes with progressively decreasing tip diameters until a homogeneous single-cell suspension was obtained. The suspension was briefly centrifuged, and the resulting cell pellet was gently resuspended in Neurobasal medium supplemented with 2% B27 and 1% GlutaMAX (all from Thermo Fisher Scientific, Waltham, MA, USA). Cells were diluted to the desired concentration and plated at the center of poly-L-lysine-coated plastic Petri dishes or MatTek dishes. Neuronal cultures were maintained at 37 °C in a humidified incubator with 5% CO_2_ in Neurobasal medium supplemented with 2% B27 and 1% GlutaMAX. Half of the culture medium was replaced every 3–4 days.

### 2.8. Immunofluorescence

Cells were fixed with 4% paraformaldehyde (PFA) in phosphate-buffered saline (PBS) for 20 min at room temperature and subsequently washed three times with PBS. Cells were then permeabilized with 0.1% Triton X-100 in PBS for 20 min and blocked with 2% bovine serum albumin (BSA) in PBS (blocking solution) for 1 h at room temperature. Primary antibodies against TMEM9A (1:50, Atlas Antibodies) and GFP (1:50, Invitrogen) were diluted in blocking solution and incubated with the cells overnight at 4 °C. Following three washes with PBS, cells were incubated for 2 h at room temperature with Alexa Fluor 568-conjugated goat anti-rabbit and Alexa Fluor 488-conjugated goat anti-mouse secondary antibodies (both from Invitrogen), diluted 1:1000 in blocking solution, then mounted using Prolong Diamond Antifade Mountant (ThermoFisher).

### 2.9. Fluorescence Colocalization Analysis

Immunofluorescence images were acquired using an IXplore™ IX85 SpinXL spinning disk confocal microscope (Evident GmbH, Hamburg, Germany) equipped with an X-Light V3 confocal spinning disk head (CrestOptics S.p.A., Rome, Italy). Images were acquired using a UPLXAPO 60×/1.42 NA oil-immersion objective lens (WD 0.15 mm; Olympus) and 470 nm and 555 nm laser lines for excitation with Alexa Fluor 488-conjugated goat anti-mouse secondary antibodies and Alexa Fluor 568-conjugated goat anti-rabbit secondary antibodies, respectively. Confocal fluorescence images were acquired using Evident CellSens software (Version 4.5, provided by Evident Europe, Hamburg, Gerrmany, 10 October 2025).

Fluorescence images were acquired using identical acquisition settings for all samples within each experiment to ensure comparability. Colocalization analysis was performed using the JACoP plugin in Fiji/ImageJ (downloaded on 10 August 2025). Prior to analysis, background subtraction was applied uniformly to all images. For each analyzed cell, a region of interest (ROI) encompassing the cellular area was defined and applied identically to the corresponding fluorescence channels. Regions devoid of cellular fluorescence and areas outside the selected ROIs were excluded from the analysis. The degree of spatial association between the two fluorescence channels was assessed by calculating the Pearson’s correlation coefficient (PCC) on a pixel-by-pixel basis within the selected ROIs.

The PCC measures the linear correlation between fluorescence intensities in the two channels and ranges from −1 to +1. Values approaching +1 indicate a strong positive correlation between the fluorescence intensities of the two channels; values close to 0 indicate little or no correlation, whereas negative values indicate an inverse relationship between the fluorescence signals. Accordingly, higher positive PCC values were interpreted as indicating a greater degree of spatial association between the two fluorescence signals. PCC values were calculated independently for individual cells and subsequently used for statistical analysis across biological replicates.

### 2.10. RNA Extraction

Adult Clcn3ven/ven mice were sacrificed by CO2 inhalation, and brain biopsies from the cortex, hippocampus, and cerebellum were immediately harvested. The tissue samples were preserved in RNA preservation reagent (RNAlater™) and stored at −80 °C. Total RNA was extracted using the Aurum™ Total RNA Mini Kit (Bio-Rad) following tissue disruption. Briefly, tissues were homogenized in Lysis buffer using a micropestle in 1.5 mL tubes and completely disrupted by shearing through a 21-gauge needle.

### 2.11. RNA Reverse Transcription and Quantitative PCR (qPCR)

Total RNA (1 µg) was reverse transcribed using the Reliance cDNA Synthesis Kit (Bio-Rad), following the manufacturer’s instructions, with oligo(dT) primers.

Quantitative PCR (qPCR) was performed on a CFX Connect Real-Time PCR Detection System (Bio-Rad) using SYBR Green qPCR Master Mix (Bio-Rad). All samples were analyzed in triplicate. For each primer pair, negative controls omitting the reverse transcriptase (-RT) and replacing the template with water (H_2_O) were included.

Relative gene expression levels were quantified using the ΔCt method, with Gapdh serving as the internal housekeeping gene control. The primer sequences utilized were identical to those described by Planells-Cases et al. [18] and are listed below.
Clcn3-FTGTAACTCACAACGGACGCCTCClcn3-RTATTGAAGCGGGGTCTTGGTTTClcn4-FGCGTCTCATCGGGTTTGCClcn4-RTTGCCACAATGCCCTCTTGTmem9a-FGTCCGCCTTACAGAAACATCATmem9a-RCTCGTACCTACACTCGCAGAGTmem9b-FCTATGGTGCGGAAACCTGCTTmem9b-RGCCCAGGATTCTCTTTATAGGGAGapdh-FTCCATGACAACTTTGGCATTGGapdh-RCAGTCTTCTGGGTGGCAGTGA

### 2.12. Statistical Analysis

All data are derived from a minimum of three independent experimental replicates, unless otherwise indicated. Results are presented as mean ± SEM (or mean ± SD, as specified in each figure legend), where n indicates the number of tested cells. Statistical comparisons across more than two groups were performed using one-way ANOVA. Statistical significance was defined as *p* < 0.05 (indicated by *) or *p* < 0.01 (indicated by **).

## 3. Results

### 3.1. TMEM9A Inhibits ClC-4 Activity in Xenopus Oocytes

While the inhibitory effect of TMEM9A on ClC-5 has been documented in detail [18], less is known about ClC-3 and ClC-4. To address this, we first tested for TMEM9A inhibition of the endosomal ClC-4 in Xenopus oocytes. Two to three days after injection of RNA encoding human ClC-4, robust outward currents could be measured using the two-electrode voltage-clamp technique, as described previously [23,25,26,27] (Figure 1A). Co-injection with TMEM9A-encoding RNA reduced currents to the level seen in uninjected oocytes (Figure 1A,B), similarly to what has been described for TMEM9B [17].

### 3.2. TMEM9A Inhibits ClC-3 Activity in Transfected HEK Cells

Since ClC-3 and ClC-4 share high structural and functional similarity, form heterodimers in vitro and in vivo [24,28], and are similarly affected by TMEM9B in Xenopus oocytes as well as transfected HEK cells [17], we selected transfected HEK cells as the expression system for ClC-3, to test for functional effects of TMEM9A, using the mouse ClC-3c splice variant as in previous studies [4,17,29,30,31]. Expressing ClC-3c alone led to robust, strongly outwardly rectifying currents and transient inward currents upon return to the holding potential of 0 mV (Figure 2A) [4,29,31]. Both components were almost completely suppressed upon the co-expression of TMEM9A (Figure 2A,B).

### 3.3. Slight Inhibition of ClC-1 by TMEM9A in Xenopus Oocytes

For ClC-1, we selected Xenopus oocytes as a highly robust expression system rather than HEK293 cells, where over-expression can easily lead to very large currents with consequent series resistance problems. Co-expression of ClC-1 with TMEM9A in Xenopus oocytes inhibited currents by ~50%, similar to what has been observed for TMEM9B (Figure 3) [17].

### 3.4. TMEM9A Does Not Affect ClC-2 in Transfected HEK Cells

For ClC-2 we selected HEK293 cells as the expression system because the resulting currents have properties that are more similar to those of endogenous ClC-2, compared to currents recorded in Xenopus oocytes [32,33]. Transfection of ClC-2 in HEK293 cells led to typical inwardly rectifying chloride currents that quickly deactivate at positive voltages (Figure 4A) [32,34,35,36,37,38]. No effect on these currents was observed upon co-transfection with TMEM9A (Figure 4A,B), similar to what has been reported for TMEM9B [17].

### 3.5. No Effect of TMEM9A on ClC-7/OSTM1 in Transfected HEK293 Cells

To achieve plasma-membrane expression of the strictly lysosomal ClC-7 transporter, an N-terminal sorting motif was mutated [12,39] and the obligatory OSTM1 subunit [11] was inserted into the expression plasmid using a self-cleavable signal peptide [40]. Since ClC-7 can be much more reliably measured in transfected HEK293 cells compared to Xenopus oocytes [12,41], we used the HEK cell system to explore possible effects of TMEM9A. Robust, slowly activating currents were seen after transfection in HEK cells (Figure 5A) [12]. Co-expression of TMEM9A had practically no effect on the currents (Figure 5A,B), as was the case also for TMEM9B [17], suggesting no potential interaction between either ClC-7 or OSTM1 and TMEM9A.

### 3.6. FLIM-FRET Analysis Shows Direct Interaction of ClC-3 and ClC-4 with TMEM9A

To determine a direct molecular interaction between CLC proteins and TMEM9A, FLIM-FRET measurements in HEK293 cells transiently expressing CLCs together with TMEM9A were performed. The CLC proteins were fused to GFP (donor) whereas TMEM9A was C-terminally tagged with mCherry (acceptor), and fluorescence lifetimes were measured as described in the Methods Section 2.5. Because FRET occurs only when donor and acceptor fluorophores are within ~10 nm apart [42], a reduction in donor lifetime provides evidence for close molecular proximity. Among the CLC family members we chose ClC-3 and ClC-4 as the relevant endosomal targets of TMEM9A, ClC-1 as a representative of the plasma membrane-localized channel CLCs, and ClC-7 to represent the ClC-6/ClC-7 branch of lysosomal/late-endosomal CLCs.

Representative phasor plots, the corresponding fluorescence lifetime values, and merged confocal fluorescence images for each experimental condition are shown in Figure 6. Cells expressing ClC-3-GFP alone displayed a donor lifetime (τ_D_) of 2.48 ± 0.01 ns, which decreased to 2.09 ± 0.04 ns upon co-expression with TMEM9A-mCherry (Figure 6A,B). Likewise, the lifetime of ClC-4-GFP was reduced from 2.41 ± 0.01 ns to 2.04 ± 0.03 ns in the presence of TMEM9A-mCherry (Figure 6C,D). In both cases, the merged fluorescence images revealed extensive signal overlap between the two proteins, consistent with the observed FRET responses. The calculated mean FRET efficiencies were 0.19 ± 0.02 for the ClC-3-GFP/TMEM9A-mCherry pair and 0.15 ± 0.02 for the ClC-4-GFP/TMEM9A-mCherry pair (Figure 6I). These findings support direct interaction of TMEM9A with both ClC-3 and ClC-4.

In contrast, co-expression of TMEM9A-mCherry with either ClC-1-GFP or ClC-7-GFP produced only minor changes in donor lifetime (Figure 6E–H). Cells expressing ClC-1-GFP alone displayed a donor lifetime of 2.53 ± 0.04 ns, which decreased to 2.34 ± 0.04 ns upon co-expression with TMEM9A-mCherry and cells expressing ClC-7-GFP alone displayed a donor lifetime of 2.56 ± 0.04 ns, which remained constant at 2.51 ± 0.04 ns upon co-expression with TMEM9A-mCherry (Figure 6E–H). The resulting mean FRET efficiencies were 0.07 ± 0.02 for ClC-1 and 0.02 ± 0.01 for ClC-7 (Figure 6I), indicating substantially weaker proximity between these proteins and TMEM9A. Taken together, these results suggest that TMEM9A preferentially associates with ClC-3 and ClC-4, whereas interactions with ClC-1 and ClC-7, if present, are considerably less pronounced, confirming the observed electrophysiological patch- and voltage-clamp characterizations (Figure 3 and Figure 5). The individual donor and acceptor confocal fluorescence images, together with the corresponding merged images, are provided in the Supplementary Materials (Supplementary Figure S1).

### 3.7. Stable Expression of Clcn3, Clcn4, Tmem9a, and Tmem9b Across Brain Regions

mRNA expression levels of Clcn3, Clcn4, Tmem9a, and Tmem9b were analyzed in the cortex, hippocampus, and cerebellum of three adult Clcn3ven/ven mice. All transcripts were found to be relatively uniformly expressed in all brain regions, with no marked differences between TMEM9A and TMEM9B, or between Clcn3 or Clcn4 (Figure 7). Clcn3 and Clcn4 were slightly more abundant in cortex compared to hippocampus or cerebellum, but the difference was not statistically significant.

### 3.8. TMEM9A Colocalization with ClC-3 and ClC-4 in Primary Neurons and HeLa Cells

Confocal imaging was performed to assess the localization of TMEM9A (red) and ClC-3 or ClC-4 transporters (green) in primary cortical neurons (Figure 8A–C) and HeLa cells (Figure 8D–I).

Colocalization between TMEM9A and CLC family proteins in fixed cell fluorescence imaging analysis was quantified using Pearson’s correlation coefficient (PCC).

In primary cortical neurons from Clcn3 ven/ven mice expressing VenusClC-3, TMEM9A immunostaining showed a moderate positive degree of colocalization with VenusClC-3, detected using an anti-GFP antibody, with a PCC mean value of 0.52 ± 0.03 (Figure 8C), indicating partial overlap of the fluorescence signals. In HeLa cells co-expressing TMEM9A and ClC-3-GFP, a stronger correlation between TMEM9A immunostaining (Alexa Fluor 568) and ClC-3-GFP fluorescence (Alexa Fluor 488) was observed, with a mean PCC of 0.73 ± 0.08 (Figure 8F).

Similarly, TMEM9A showed a positive correlation with ClC-4 in transfected HeLa cells showing a PCC mean value of 0.65 ± 0.04 (Figure 8I).

Overall, these findings support a spatial association of TMEM9A with intracellular compartments containing vesicular ClC-3 and ClC-4 transporters.

## 4. Discussion

The aim of this study was to determine whether TMEM9A shares the interaction specificity and inhibitory properties previously described for TMEM9B and to define its selectivity within the CLC protein family. In particular, we examined whether TMEM9A specifically regulates the neuronal endosomal Cl^−^/H^+^ exchangers ClC-3 and ClC-4 or whether it also affects other CLC proteins. In addition, we sought to corroborate the functional data by direct interaction measurements and to assess whether the expression pattern of TMEM9A is compatible with a physiological role in the nervous system.

Our results show that TMEM9A strongly inhibits ClC-3 and ClC-4 currents, whereas it has no detectable effect on ClC-2 or ClC-7 and only a modest effect on ClC-1. FLIM-FRET experiments closely mirror these functional observations, revealing clear interactions with ClC-3 and ClC-4 but not with ClC-1 or ClC-7. Furthermore, endogenous Tmem9a and Tmem9b are broadly expressed throughout the mouse brain together with Clcn3 and Clcn4, and TMEM9A partially colocalizes with ClC-3 in primary neurons and with both overexpressed ClC-3 and ClC-4 in transfected cells.

The present findings extend our previous work on TMEM9B [17]. In that study, TMEM9B was identified as the first accessory protein capable of inhibiting ClC-3 and ClC-4. Here we show that TMEM9A exhibits an almost identical selectivity profile. Both proteins strongly inhibit ClC-3 and ClC-4, while leaving ClC-2 and ClC-7 unaffected. The small reduction in ClC-1 currents is also similar for the two proteins and is accompanied by only weak FLIM-FRET signals. It might be interesting to test whether the interaction with ClC-1 has any physiological role.

While the present work was in progress, two studies considerably advanced the understanding of TMEM9A proteins. Planells-Cases et al. [18] demonstrated that TMEM9A and TMEM9B are required in vivo for normal expression of ClC-3, ClC-4 and ClC-5. Simultaneous deletion of both genes resulted in embryonic or perinatal lethality together with a marked reduction in all three transporters, establishing TMEM9A and TMEM9B proteins as bona fide β-subunits of the endosomal CLC branch. Independently, Schrecker et al. [19] determined the cryo-EM structure of the ClC-3/TMEM9A complex and showed that TMEM9A binds at the cytoplasmic side of the transporter, where it blocks access to the chloride permeation pathway. In addition, binding is stabilized by PtdIns(3,5)P2, suggesting that transporter inhibition is integrated with the lipid composition of endosomal membranes.

Our results fit well into this emerging picture. Although we did not address the structural basis of inhibition, the excellent agreement between electrophysiology and FLIM-FRET strongly suggests that inhibition results from direct binding of TMEM9A to ClC-3 and ClC-4 rather than from indirect effects on expression or trafficking. Conversely, the absence of measurable FRET for ClC-2 and ClC-7 is fully consistent with the lack of functional inhibition. Thus, both the functional and interaction data support a high degree of specificity of TMEM9A and TMEM9B for the neuronal endosomal CLC transporters.

The systematic comparison with other members of the CLC family further emphasizes this specificity. CLC accessory proteins are generally highly selective. Barttin is required for ClC-K channels, but not for other CLC proteins [7]; OSTM1 specifically stabilizes ClC-7 [11], and GlialCAM regulates ClC-2 in glial cells [14,43]. TMEM9A and TMEM9B now appear to define an analogous accessory protein system for the ClC-3/-4/-5 branch. Such specificity probably reflects structural determinants that are conserved within this subgroup but absent from the plasma membrane channels and from ClC-7.

The qPCR experiments revealed broad and largely overlapping expression of Tmem9a, Tmem9b, Clcn3 and Clcn4 throughout the adult brain. Although these measurements do not provide cellular resolution, they are compatible with the extensive functional redundancy suggested by the knockout phenotype reported by Planells-Cases et al. Indeed, the relatively mild phenotype of the individual knockouts compared with the severe phenotype of the double knockout suggests that the two proteins can compensate for each other to a large extent. Whether they are expressed in identical neuronal populations or display subtle cell-type-specific differences remains to be determined.

The immunofluorescence experiments provide additional support for a physiological interaction. Colocalization between endogenous TMEM9A and Venus-tagged ClC-3 in primary neurons was moderate, whereas substantially higher overlap was observed after heterologous overexpression in HeLa cells. This difference is expected.

The moderate colocalization observed in primary neurons compared with the higher values obtained in transfected HeLa cells likely reflects differences in protein expression levels and the intrinsic complexity of sorting and protein trafficking in the two experimental systems. There might be specialized sorting machinery differences between neurons and HeLa cells, and endogenous endosomal compartments are heterogeneous and dynamic, and antibodies may not have equal access to all compartments in neurons. In contrast, overexpression in heterologous systems like HeLa cells might tend to enrich proteins within the same trafficking pathway and therefore increase the apparent degree of colocalization. Importantly, the neuronal experiments demonstrate that endogenous TMEM9A and ClC-3 are present within overlapping intracellular compartments, consistent with the possibility of direct interaction in vivo.

An interesting question that remains unresolved is whether TMEM9A and TMEM9B are completely interchangeable or whether they fulfill partially distinct physiological functions. The similarity of their effects in heterologous expression systems suggests that they regulate transporter activity through essentially the same mechanism. However, differences in expression levels, intracellular localization, developmental regulation or affinity for individual transporters could still confer distinct physiological roles, as CLC β-subunits or accessory proteins functions can extend to trafficking, membrane targeting, gating modulation, stability, and degradation controls. Likewise, it is currently unknown whether individual transporter dimers bind either TMEM9A or TMEM9B exclusively or whether more complex regulatory assemblies exist. It is also unknown whether pathological CLC variants like the wild types have similar responsiveness to and resistance to TMEM9A and TMEM9B regulation; variants responsive to TMEM9A might be resistant to TMEM9B, and vice versa. These findings could help identify regulatory differences between TMEM9A and TMEM9B.

Limitations of the study: Baseline expression of endogenous CLC and TMEM9 proteins in the heterologous expression systems was not quantified, and scrambled RNA injection controls were not performed. Although we consider these controls unlikely to affect the interpretation of our results, we acknowledge that their absence limits the assessment of potential contributions from endogenous expression and nonspecific effects of RNA injection.

## 5. Conclusions

In summary, the present study shows that TMEM9A closely resembles TMEM9B with respect to both interaction specificity and functional inhibition of endosomal CLC wild types. Together with the recent genetic and structural studies, our results support the conclusion that TMEM9 proteins constitute accessory β-subunits of the endosomal ClC-3/-4/-5 transporters. These findings establish another parallel between the endosomal CLC transporters and other members of the CLC family, whose physiological function likewise depends on dedicated accessory proteins. Further work will be required to understand how TMEM9A and TMEM9B cooperate in different cell types and how their interaction with CLC transporters is regulated under physiological and pathological conditions.

## Figures and Tables

**Figure 1:**
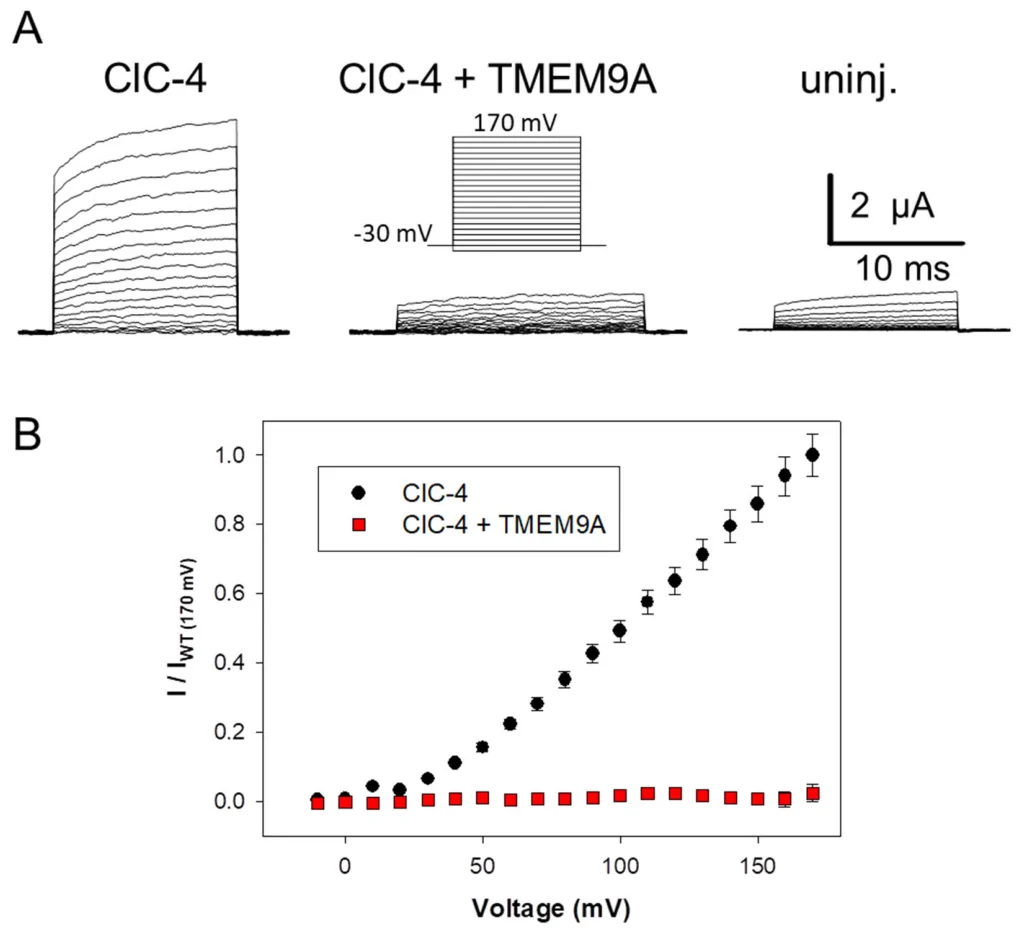
Strong inhibitory effect of TMEM9A on ClC-4 activity in Xenopus oocytes. (**A**) Representative current traces recorded from Xenopus oocytes injected with ClC-4, ClC-4 + TMEM9A, or uninjected (control) oocytes, evoked by the voltage-clamp protocol shown in the inset. (**B**) Current–voltage (I–V) relationships showing the average normalized current amplitudes for ClC-4 (black dots) compared with ClC-4 co-injected with TMEM9A (red squares). Of note, the I–V plot reflects the subtraction of average currents measured in uninjected oocytes from the same batches. In some instances, error bars are smaller than the symbol size. Data are expressed as mean ± SD with n = 4 batches with ≥4 oocytes for each combination and batch.

**Figure 2:**
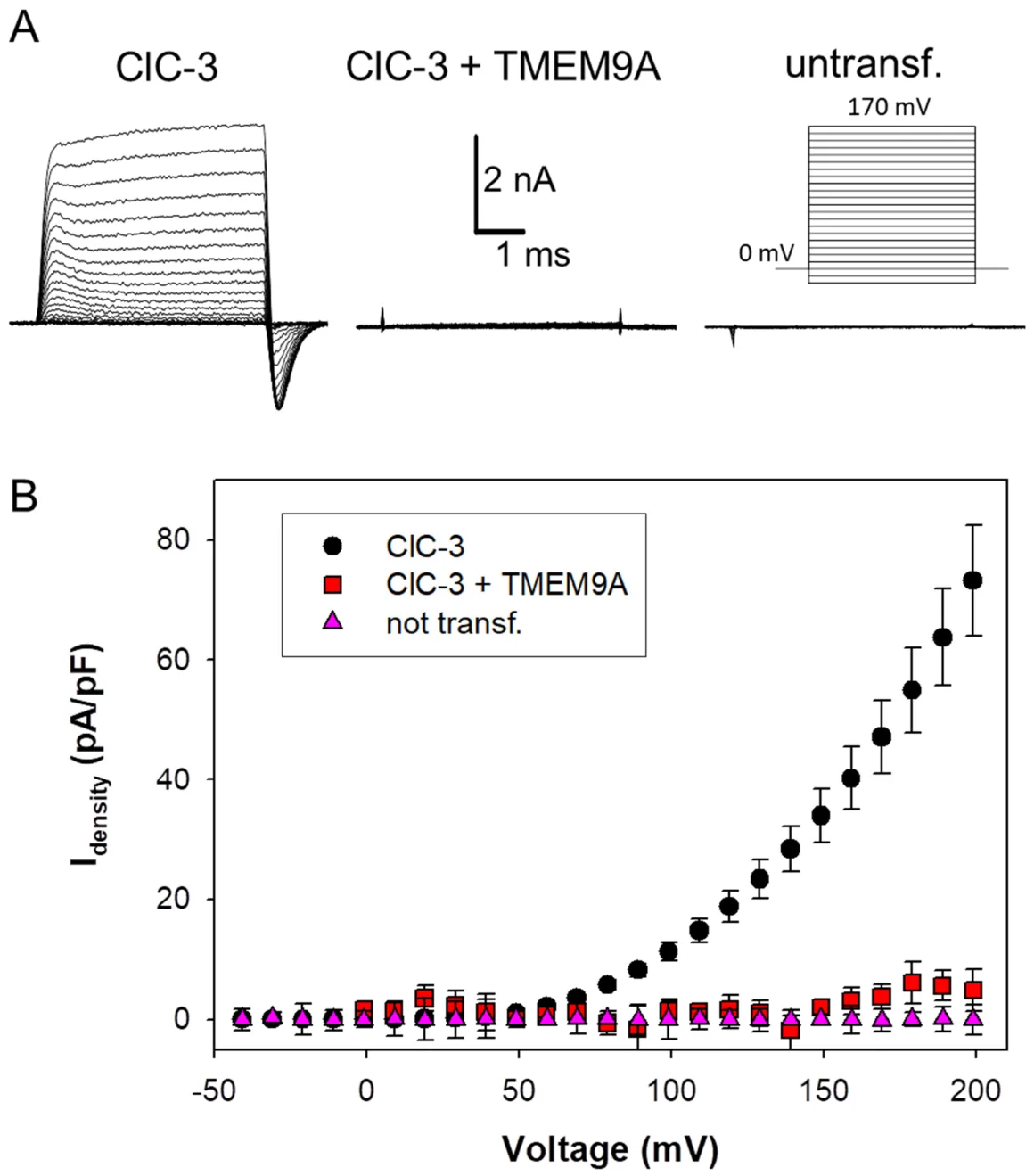
Pronounced inhibitory effect of TMEM9A on the ClC-3c splice variant activity in HEK293 cells. (**A**) Representative current traces mediated by ClC-3c alone (left), co-expressed with TMEM9A (middle), or from an untransfected cell (right). The voltage-clamp protocol used to evoke the currents is shown. (**B**) Current–voltage (I–V) relationships of steady-state currents for ClC-3c alone (black dots) compared with ClC-3c co-expressed with TMEM9A (red squares) and untransfected cells (pink triangles). Data are presented as mean ± SD, n = 7 each.

**Figure 3:**
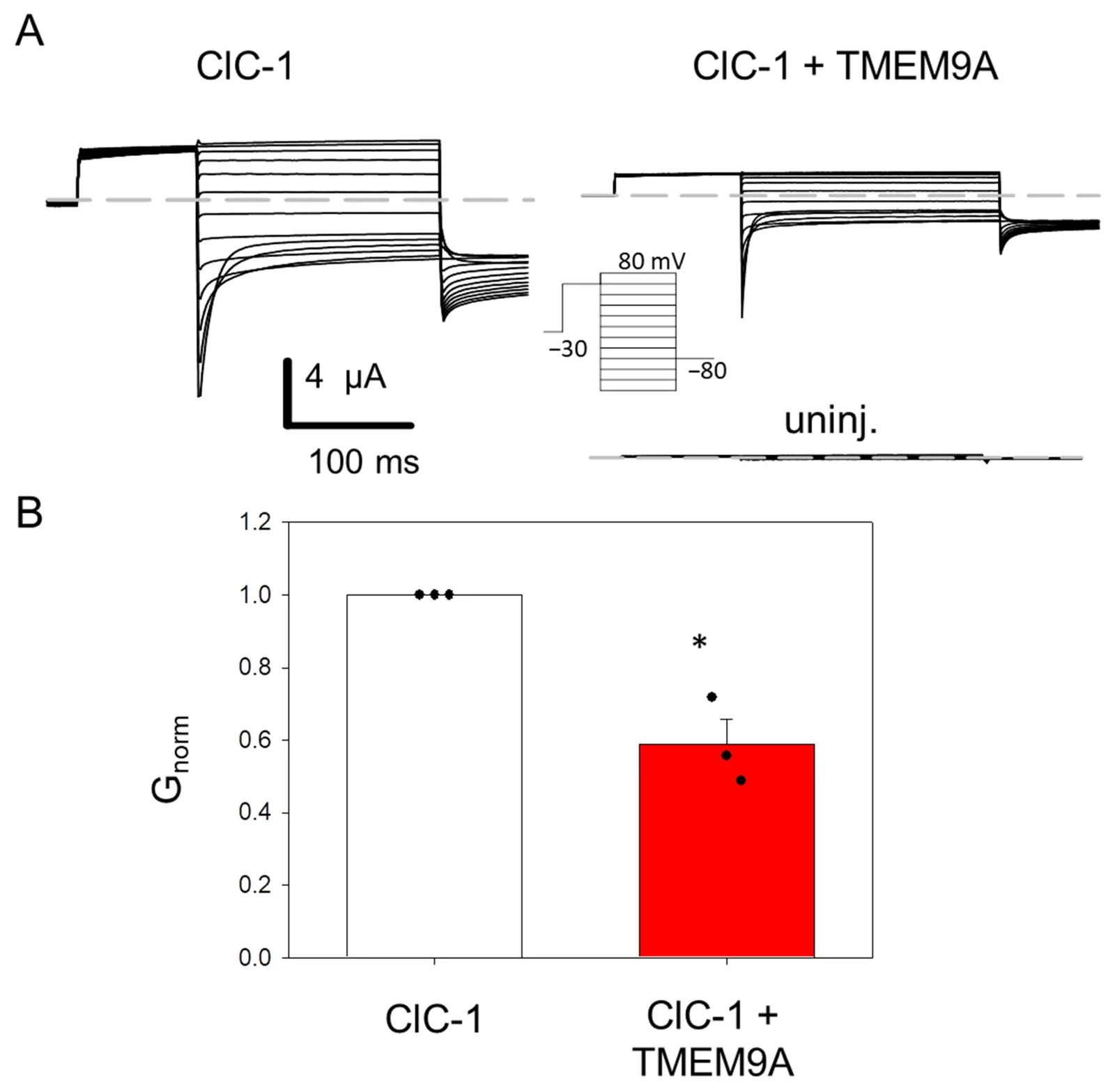
Modest inhibitory effect of TMEM9A on ClC-1 activity in Xenopus oocytes. (**A**) Representative current traces recorded from Xenopus oocytes injected with ClC-1 (left), ClC-1 co-injected with TMEM9A (right, top), and from an uninjected oocyte (right, bottom), evoked by the voltage-clamp protocol shown in the inset. (**B**) The column graph shows normalized conductance (see Methods) of ClC-1 (white column) compared with ClC-1 + TMEM9A (red column). Dashed line indicates zero-current level. Data are expressed as mean ± SD, n = 3 batches of oocytes with >4 oocytes for each batch and condition. * indicates *p* < 0.05.

**Figure 4:**
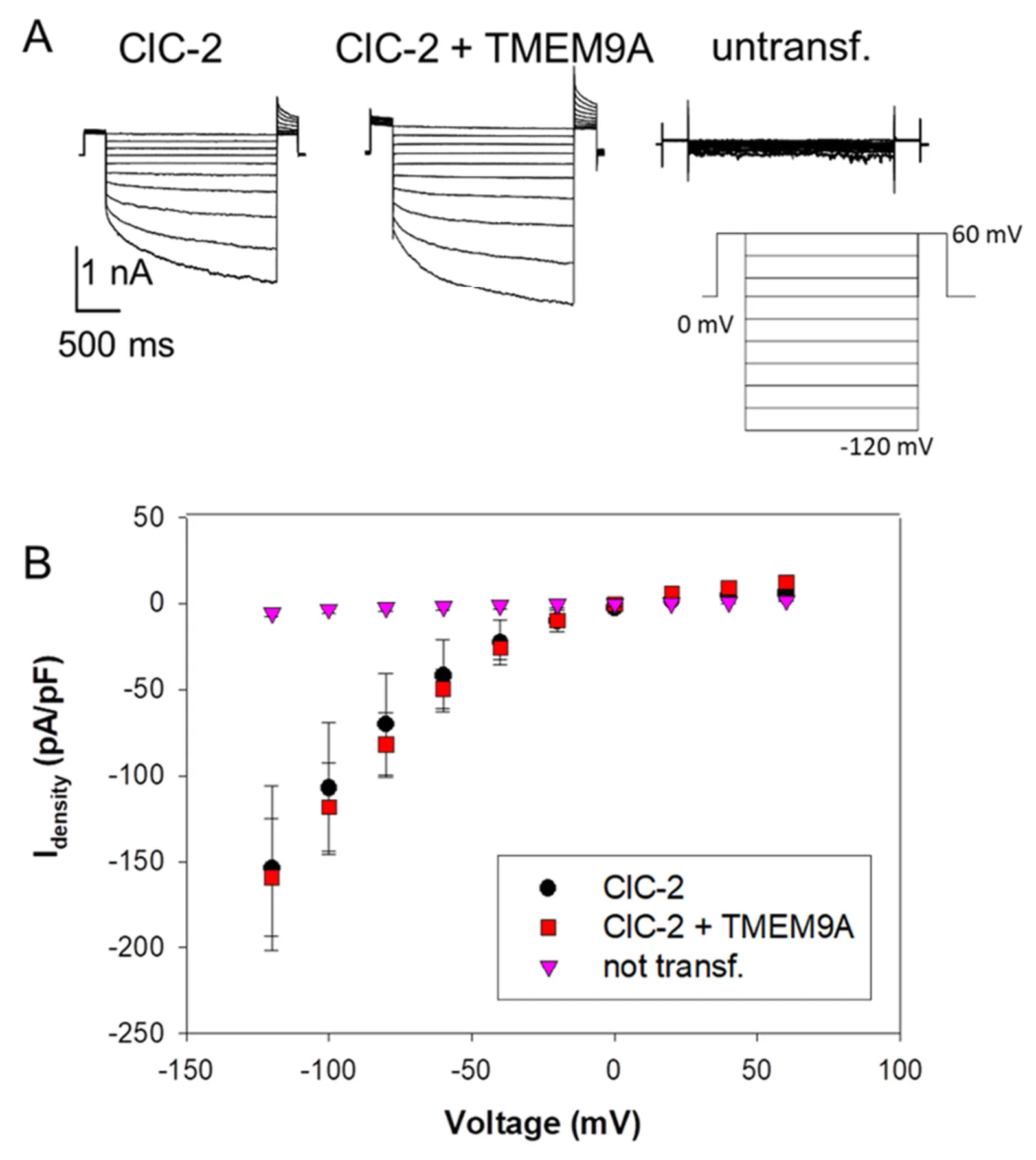
No effect of TMEM9A on ClC-2 in HEK293 cells. (**A**) Representative current traces mediated by ClC-2 alone (left), co-expressed with TMEM9A (middle), and from an untransfected cell (right). The voltage-clamp protocol used to evoke the currents is shown. (**B**) Current–voltage (I–V) relationships of steady-state currents for ClC-2 alone (black dots) compared with ClC-2 co-expressed with TMEM9A (red squares), and untransfected cells (pink triangles). Data are presented as mean ± SD with n = 7 (ClC-2 alone) and n = 8 (ClC-2 + TMEM9A).

**Figure 5:**
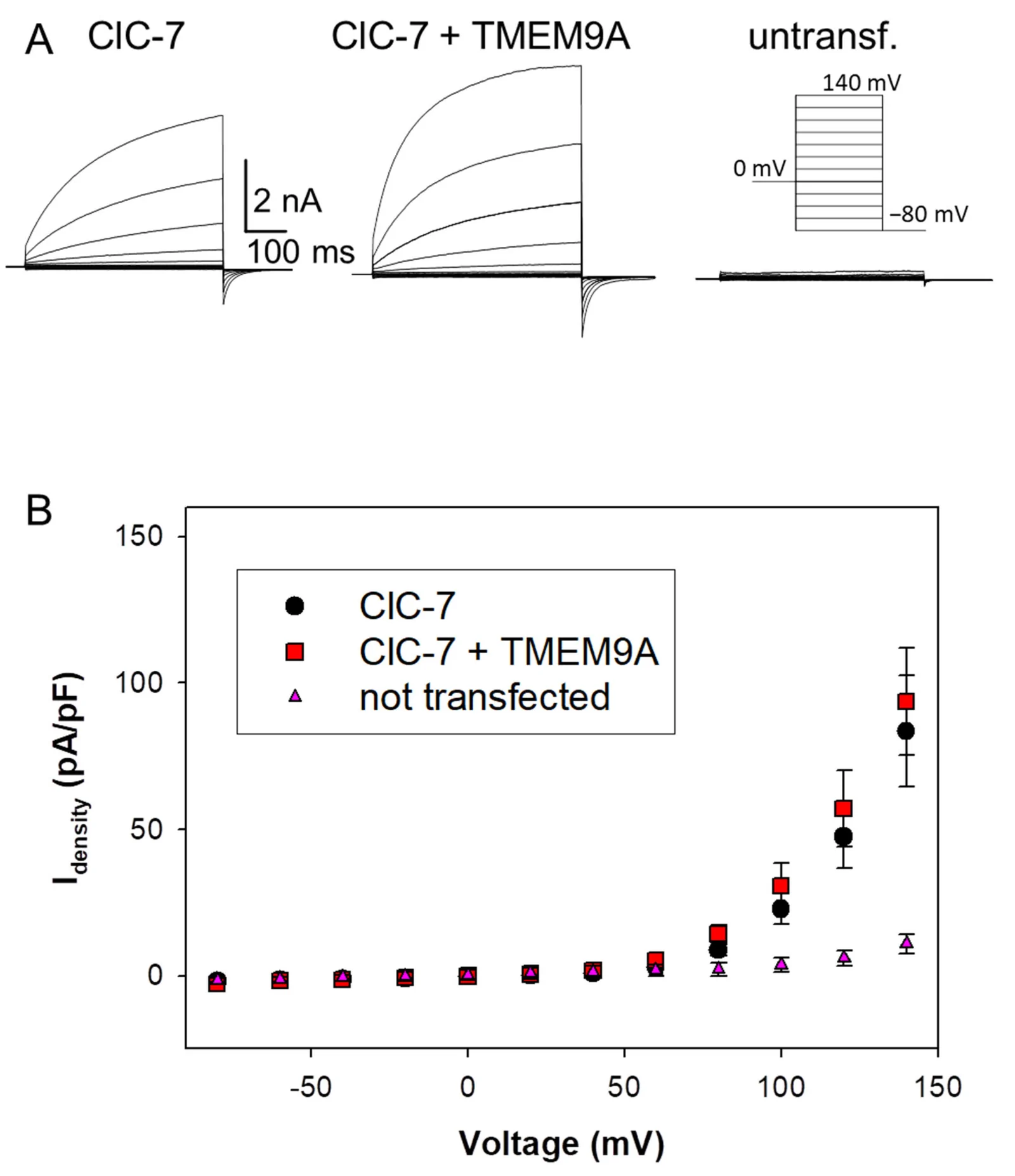
No effect of TMEM9A on ClC-7/OSTM1 in HEK293 cells. (**A**) Typical current traces mediated by ClC-7 alone (left), co-expressed with TMEM9A (middle), and from an untransfected cell (right). The voltage-clamp protocol used to evoke the currents is shown. (**B**) Current–voltage (I–V) relationships of steady-state currents for ClC-7 alone (black dots) compared with ClC-7 co-expressed with TMEM9A (red squares), and untransfected cells (pink triangles). Data are presented as mean ± SD, n = 5 for each condition.

**Figure 6:**
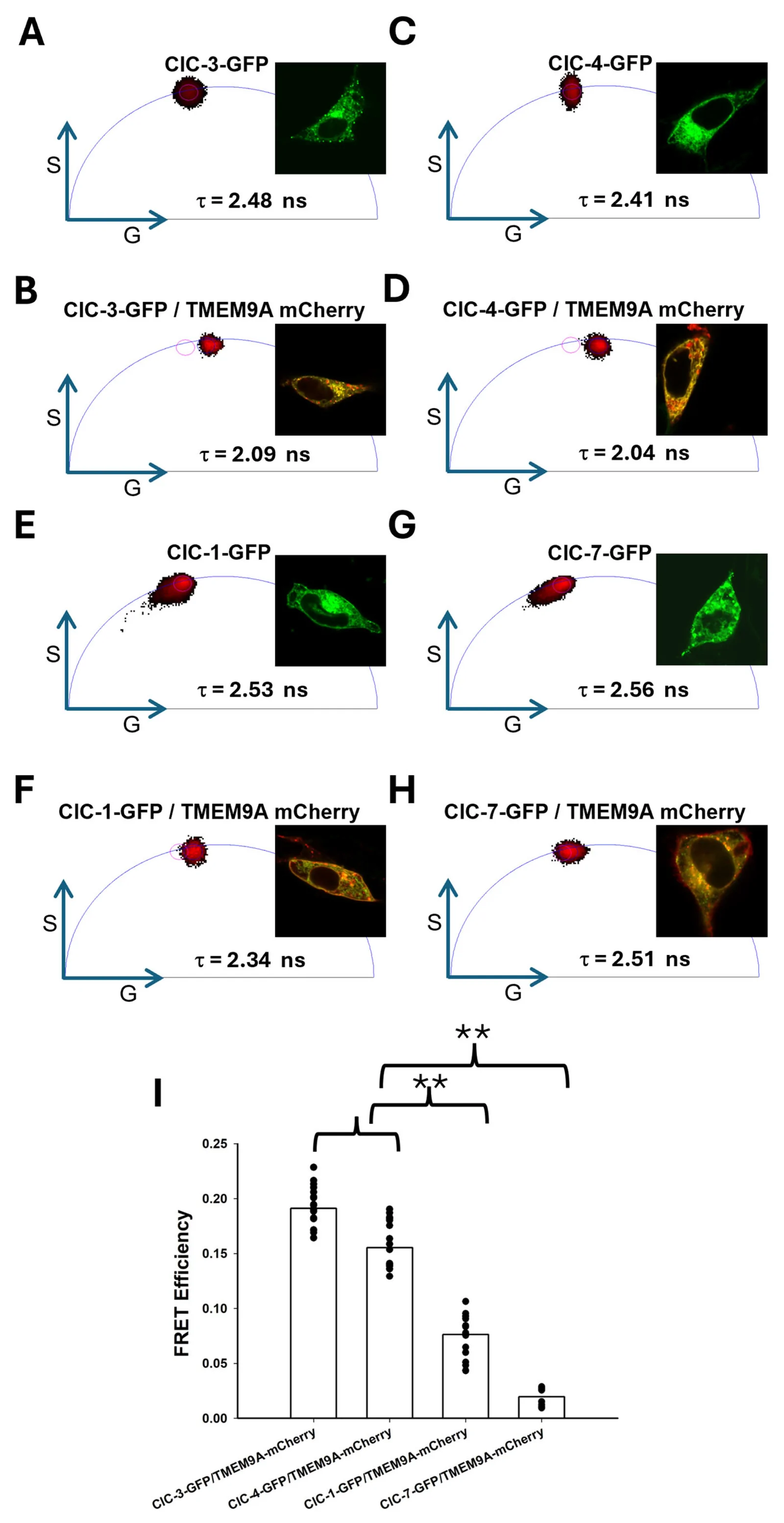
FLIM-FRET analysis of CLC protein interaction with TMEM9A in HEK293 cells. (**A**) Representative phasor distribution, corresponding fluorescence lifetime, and confocal image of HEK293 cells expressing ClC-3-GFP. (**B**) Phasor plot, measured lifetime, and representative merged confocal image obtained from cells co-expressing ClC-3-GFP and TMEM9A-mCherry. In this panel, and in (**D**,**F**,**H**), the purple marker denotes the donor fluorophore phasor position in the absence of FRET, and the blue marker indicates the donor phasor coordinates measured in the presence of the acceptor. (**C**) Phasor analysis, lifetime determination, and representative confocal image of cells transfected with ClC-4-GFP. (**D**) Corresponding phasor plot, lifetime value, and merged fluorescence image from ClC-4-GFP/TMEM9A-mCherry co-transfected cells. (**E**) Representative phasor map, fluorescence lifetime, and confocal image of HEK293 cells expressing ClC-1-GFP. (**F**) Phasor distribution, associated lifetime, and representative image of cells co-expressing ClC-1-GFP and TMEM9A-mCherry. (**G**) Phasor plot analysis, lifetime measurement, and representative image of cells expressing ClC-7-GFP alone. (**H**) Corresponding phasor plot, fluorescence lifetime, and representative confocal image of ClC-7-GFP/TMEM9A-mCherry co-transfected cells. (**I**) Quantitative comparison of FRET efficiencies among the different experimental groups. Data are expressed as mean value ± SE. Statistical significance: ** *p* < 0.01.

**Figure 7:**
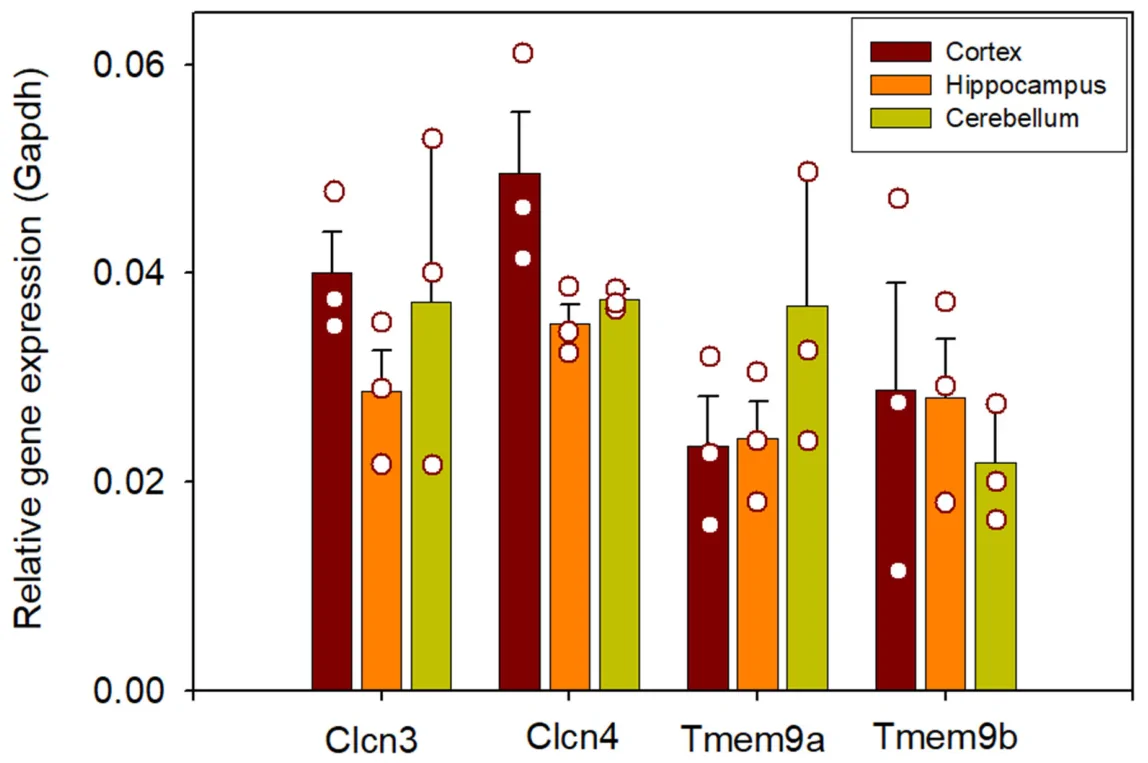
Gene expression profiles of Clcn3, Clcn4, Tmem9a, and Tmem9b in specific mouse brain regions. Relative mRNA fold expression was determined via qPCR, using Gapdh as the housekeeping gene. Data are grouped by target genes to evaluate relative expression trends across the cortex, hippocampus, and cerebellum, thereby avoiding potential confounding factors associated with differences in primer amplification efficiency among targets. Data are presented as mean ± SEM (n = 3 biological replicates, with 3 technical replicates each).

**Figure 8:**
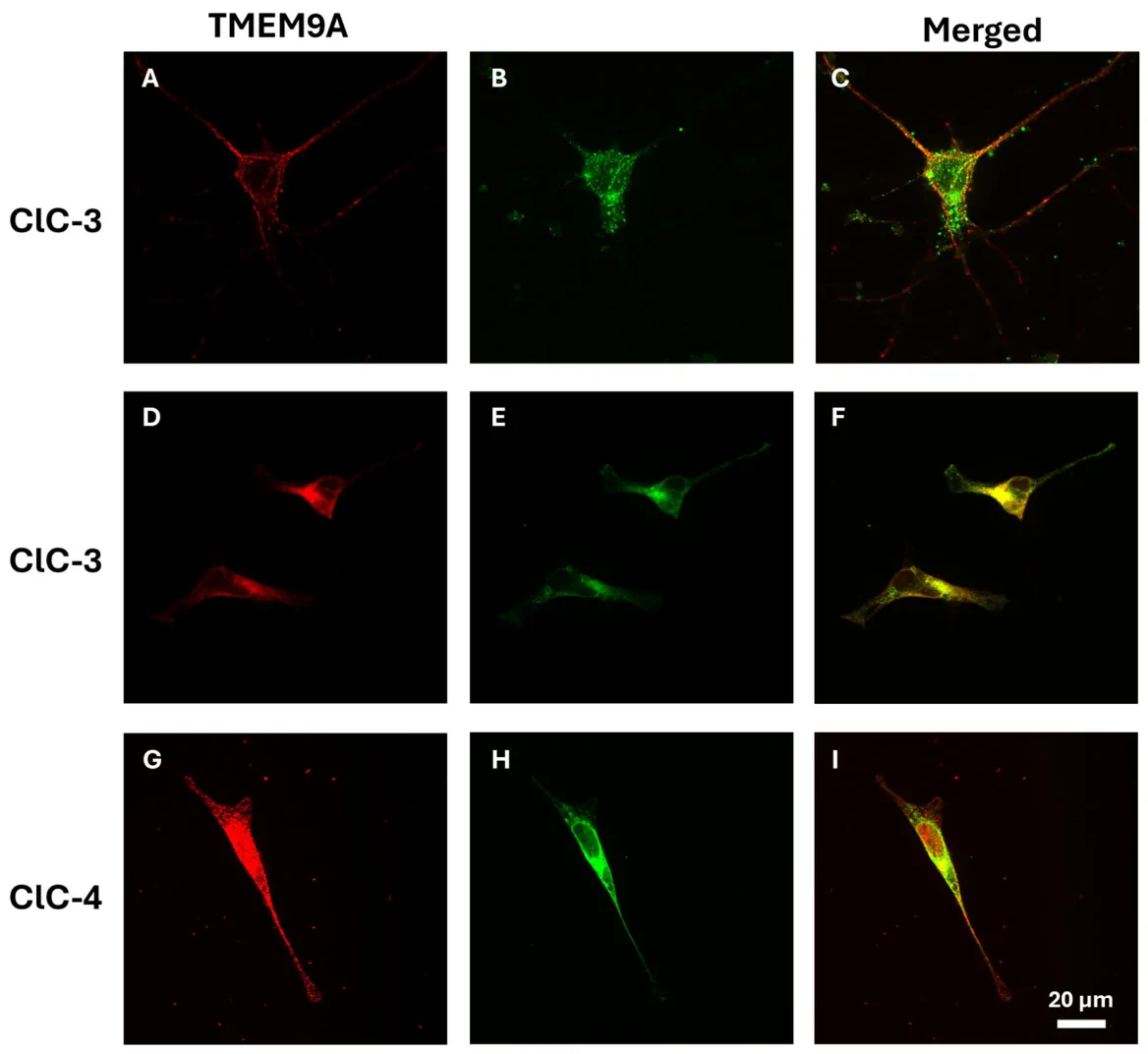
Colocalization of TMEM9A with ClC-3 and ClC-4 in primary neurons and HeLa cells. (**A**,**B**) Representative confocal images of primary cortical neurons from Clcn3 ven/ven mice expressing VenusClC-3, immunostained for TMEM9A and GFP to detect ClC-3. (**C**) Merged confocal image of panels A and B showing partial overlap of the fluorescence signals. (**D**,**E**) Representative confocal images of HeLa cells co-expressing TMEM9A and ClC-3-GFP immunolabeled for TMEM9A with Alexa Fluor 568 and for ClC-3-GFP with Alexa Fluor 488. (**F**) Merged confocal image of panels D and E showing substantial overlap of the fluorescence signals. (**G**,**H**) Representative confocal images of HeLa cells co-expressing TMEM9A and ClC-4. (**I**) Merged confocal image of panels (**G**,**H**), showing overlap of the fluorescence signals. Yellow in the merged images indicates overlapping fluorescence signals. At least n = 5 cells from 3 independent biological experiments were analyzed for each experimental condition.

## Data Availability

Primary data shown in the Figures will be made available upon reasonable request.

## Supplementary Materials

The following are available online at https://www.mdpi.com/article/10.3390/cells15211932/s1, Figure S1: Representative separate confocal images of HEK293 cells.

## Abbreviations

The following abbreviations are used in this manuscript:

FLIM	Fluorescence lifetime imaging
FRET	Förster resonance energy transfer
PBS	Phosphate-buffered solution
