# Supplementary material for: Specific Interaction of TMEM9A with Neural Endosomal Antiporters ClC-3 and ClC-4
Source: Cells. 2026 Oct 24;15(21):1932. doi: 10.3390/cells15211932 (PMC13761452; doi:10.3390/cells15211932)
Supplement: Supplementary file 1 [file cells-15-01932-s001.zip › cells-4610753-supplementary.pdf]

Supplementary Figure

Supplementary Figure S1

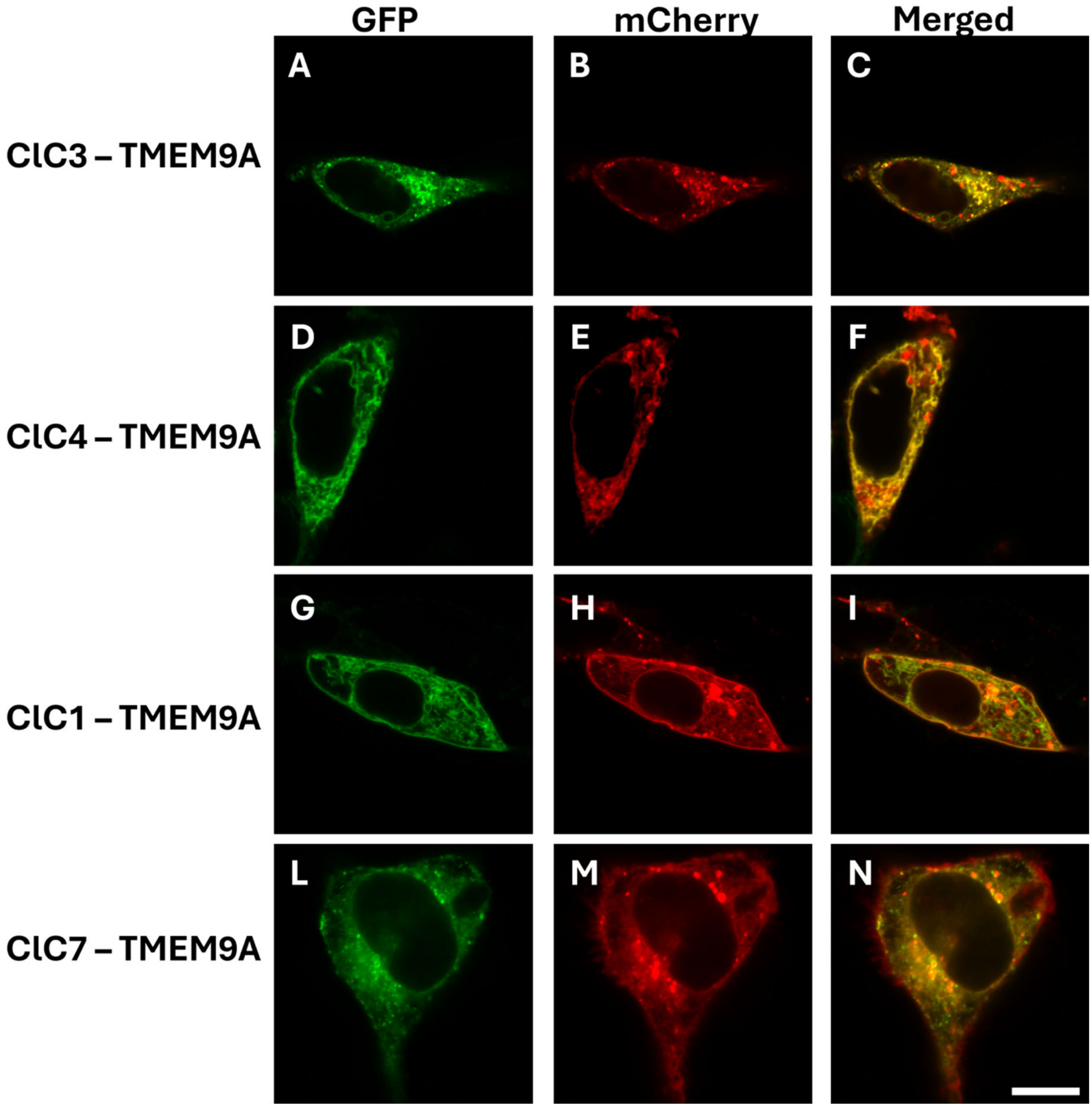

Supplementary Figure S1. Representative separate confocal images of HEK293 cells co-expressing ClC-3-GFP and TMEM9A-mCherry together with the merged image (panels A,B,C). Panels D-N show similar representative images for ClC-4-GFP/TMEM9A-mCherry, ClC-1-GFP/TMEM9A-mCherry and ClC-7-GFP/TMEM9A-mCherry, respectively. Scale bar is 10 µm.
